# Sensory Complexity: From Sensory Measurement to Consumption Behavior

**DOI:** 10.3390/foods12010029

**Published:** 2022-12-21

**Authors:** Begoña Panea, Francisco Javier Mesías, Luis Guerrero

**Affiliations:** 1 Animal Science Area, Agrifood Research and Technology Centre of Aragon (CITA), Avda. Montañana 930, 50059 Zaragoza, Spain; 2 Agrifood Institute of Aragon-IA2 (CITA-Zaragoza University), Miguel Servet 177, 50013 Zaragoza, Spain; 3 Department of Economics, University of Extremadura School of Agricultural Engineering, Avenida Adolfo Suárez, S/N, 06007 Badajoz, Spain; 4 Institute of Agrifood Research and Technology IRTA, 08140 Caldes de Montbui, Barcelona, Spain

Sensory analysis is a multidisciplinary field that includes the measurement, interpretation, and understanding of human responses to the sensory properties of products and it is essential to explore consumer behavior. Understanding consumer preferences is key to the development of the food industry, but consumer behavior is complex because of the diversity of factors influencing food choice. The attitude of the consumer results from the multiple attributes perceived of a product and depends on the importance assigned to each of them. This perception has the following three dimensions: a cognitive (knowledge), an affective (attitude, feelings) and a conative (behavior, purchase intention, and actual purchase) dimension. As a consequence, external cues such as the availability of resources, nutritional value, sensory quality or socio-environmental impact of the production, as well as internal cues, such as age, gender, educational level, incomes or cultural background, will affect both consumers’ behavior. 

Consumer habits depend on availability of resources. In Central and West Africa, plantain is a staple food; however, there is limited understanding of cooking methods, consumer preference, and willingness to pay for plantain and plantain-based products. The paper entitled “Consumer Preferences and Socioeconomic Factors Decided on Plantain and Plantain-Based Products in the Central Region of Cameroon and Oyo State, Nigeria” [1] aims to investigate these items in Cameroon and Nigeria. A household survey sample of 454 Cameroonian consumers and 418 Nigerian consumers was conducted. The results showed that the trend in consumption of all plantain-based products was constant in Cameroon but increased in Nigeria. The most important factor influencing Cameroonian consumers’ choice of plantain and its products was taste, while the nutrition trait influenced Nigerian consumers. Both Cameroonian and Nigerian consumers considered packaging, the location of produce, and size and quantity as the least important factors. In addition, socioeconomic characteristics were significant determinants of consumers’ choice to consume plantain and its products. Gender significantly influenced (*p* < 0.05) taste, while nutrition was significantly driven (*p* < 0.05) by education and annual income. Household size played a significant role (*p* < 0.05) in consumers’ choices when the price was considered. 

Food consumption is also associated with environmental sustainability. The study entitled “Spatial-Temporal Footprints Assessment and Driving Mechanism of China Household Diet Based on CHNS” [2] applied the China Health and Nutrition Survey (CHNS) database to quantitatively study the spatial-temporal analysis of multiple footprints of household food consumption. The results showed that, except land footprint (LF), the other four types of footprints all decreased at varying degrees; the water footprint (WF), carbon footprint (CF), nitrogen footprint (NF) and energy footprint (EF) decreased by 18.24%, 17.82%, 12.03% and 20.36%, respectively, from 2000 to 2011. The study shows that mainly nutrient intake, but also household attributes, educational level and health conditions, were significantly correlated with multiple footprints, and that the current Chinese dietary pattern would cause excessive resource consumption. 

The study entitled “Consumer Attitudes toward Consumption of Meat Products Containing Offal and Offal Extracts” also considered sustainability [3], since the development of food products containing offal and offal extracts could be part of the solution to the upcoming demand for animal protein. This study was conducted in Spain. Consumers’ perceptions were evaluated by means of focus group discussions and a survey (*n* = 400). The theory of planned behavior was used to examine consumer attitudes. Results indicated that nutritional properties, environmental sustainability, and affordability were the main drivers, while sensory attributes, low frequency consumption, and a perceived higher content of undesirable compounds were the main barriers. Three segments were identified according to beliefs: those in favor of these products, those that were health and environmentally conscious, and those who were reluctant about them. Attitude was the most important predictor of behavioral intention regarding the global model, while the social component (subjective norm) was significant for two of the identified segments, emphasizing the relevance of the social component for acceptability.

The perception of a product depends on the habits and backgrounds of each country. The aim of the paper entitled “Study on the Lamb Meat Consumer Behavior in Brazil” [4] was to identify the profile of Brazilian lamb meat consumers; in Brazil, the consumption of lamb still remains below that of other meat livestock. Therefore, a survey on consumer habits and preferences regarding food purchases and consumption habits, their preferences in relation to the quality attributes of lamb meat, and sociodemographic characterization was performed. The following three consumer profiles were identified: traditional, interested, and disinterested, and a fourth group was considered independent but could not be described. Among lamb meat consumers, men with a higher income seemed to be more frequent consumers than the others, and the intrinsic characteristics of meat quality, especially color and freshness, were the most important at the time of purchase. 

On the other hand, the study entitled “The Implications of COVID-19 on Chinese Consumer Preferences for Lamb Meat” [5] assessed if Chinese consumer attitudes towards a range of lamb attributes (such as origin, food safety, appearance, taste, price), and their opinions of New Zealand lamb (9- and 7-point Likert scales, respectively), had changed since the outbreak of COVID-19. The same survey was carried out in Shanghai and Beijing pre- (December 2018) and post-COVID-19 (November 2020), ~9 months after China’s initial outbreak, with 500 and 523 consumers, respectively. There were minimal differences in Chinese consumer ratings between December 2018 and November 2020 for different lamb attributes and opinions of New Zealand lamb. This study suggests COVID-19 has enhanced some food-safety-related behaviors but had fewer effects on Chinese opinions and preferences for New Zealand lamb attributes. 

In Argentina, color and intramuscular fat are the main attributes of raw beef quality; however, it is necessary to clarify how consumers use them, to establish different marketing strategies. Then, the study entitled “Color and Marbling as Predictors of Meat Quality Perception of Argentinian Consumers” [6] conducted an online survey in Argentina. It collected information on socio-demographic characteristics, purchase and consumption habits and beliefs, showing pictures related to color, marbling and the amount of fat. Regarding consumer beliefs, 90% of the respondents agreed with the sentence, “The two main characteristics defining beef quality at purchase time are meat color and marbling”. Socio-demographic characteristics affected purchase habits and beliefs; they also affected perceptions about meat color and marbling. It was possible to build three consumer groups for future marketing strategies, namely “hedonic” focused on a pleasing sensory experience, “appearance” prioritizes the visual aspects, and the “health-conscious” consumers were interested in their healthy nutrition.

The study entitled “Profile and Product Knowledge Affect the Usefulness of a Quality Label as a Tool to Differentiate a Product: A Chilean Survey” [7] was conducted to establish if consumers recognise quality labels and associate them with specific characteristics. An online survey was conducted to investigate whether Chilean consumers knew about Novillo de Osorno, for which a quality label is being developed. The survey was divided into the following five blocks: lifestyles, meat consumption and purchase habits, meat choice behavior, knowledge about Novillo de Osorno, and consumers’ socio-demographic information. The place of residence and consumer gender, age, or income were important cues in defining consumers’ lifestyles, meat consumption and purchase habits. Respondents could be grouped into the following three main groups: 1. younger people: urban with medium-high incomes, which search only for pleasure; 2. foodies uninvolved and females uninvolved: females with the highest income level that chose food for nutritional reasons; and 3. traditional people: men older than 55 with low incomes, living in the Northern areas and interested in taste and in the meat’s origin. Nearly 60% of respondents had never heard about Novillo de Osorno. 

The study entitled “Investigating Italian Consumer Preferences for Different Characteristics of Provolone Valpadana Using the Conjoint Analysis Approach” [8] had two main objectives. First, they aimed to estimate consumer preferences for Italian cheese (Provolone Valpadana) with respect to several attributes and levels, such as price, origin certification, production system, ‘free from’ labelling, and brand. Second, they aimed to identify consumer clusters with similar preferences for various cheese characteristics. Preferences were estimated using the conjoint analysis method. Then, a cluster analysis was used to classify consumers into different (three) clusters followed by a market simulation. In all three clusters, the attribute most preferred by Italian consumers was the brand of the cheese: consumers preferred to purchase Provolone cheese which had the lowest price, bearing a European Union (EU) quality certification, produced organically, and non-lactose-free. 

The knowledge of a product determines its acceptability, and, in this sense, traditional products tend to be more widely accepted. But what is a traditional product? This is the question raised in the study “What Turns a Product into a Traditional One?” [9] The concept of a Traditional Food Product (TFP) is made up of seven dimensions. However, it is not yet clear what the contributions of these dimensions are to the perception of the traditional image of a specific product. In addition, the effects of constructs such as habit, product involvement and objective and subjective knowledge on the traditional character of a product have not been explored either. The aims of this work were to evaluate the influence of the dimensions of the traditional food concept on the perception of mezcal and tequila and to understand consumers’ perception of the traditional character of the beverages through their segmentation characteristics. Eight hundred consumers were surveyed in four Mexican cities. A questionnaire was designed to assess the constructs, TFPs’ dimensions, sociodemographic information and consumption patterns. Results showed that the dimensions of the traditional concept provided a better understanding of the traditional character of the product, as well as their individual relevance, thereby showing that frequent consumption is not always linked to the traditional character of a product. Three clusters were obtained for both products based on the assessed dimensions of the traditional concept. The presence of the segments revealed variations in the contribution of the different dimensions to the concept of “traditional”. Geographic location, special dates and sensory dimensions are determinants in the traditional perception of both beverages, which is useful to design effective strategies to promote rational and responsible consumption.

In contrast, neophobia is a barrier to taste new products. The study entitled “Flavor Profiling by Consumers Segmented According to Product Involvement and Food Neophobia” [10] focused on differences in discriminative ability and perceptual sensitivity according to levels of product involvement or food neophobia during the intensity rating of sensory attributes in consumer profiling. Consumers (*n* = 247) rated the intensity of attributes for seven flavored black tea drinks and completed the Food Neophobia Scale and the Personal Involvement Inventory measuring product involvement with a flavored black tea drink. In the higher product involvement (IH) group and the lower food neophobia (NL) group, the number of sensory attributes representing the sample effect and of subsets discriminating the samples were greater, and the greater total variance of the samples was explained. The higher the product involvement or the lower the food neophobia, the greater the differentiation in characterizing samples with more attributes in terms of the intensity ratings. Interestingly, the high food neophobia (NH) group showed a lower active performance compared to the NL group during the sensory evaluation overall; however, the NH group was more concerned about unfamiliar attributes and samples. The results implied that the positive attitude resulting from high product involvement and low food neophobia may induce more active behavior and better performance during the sensory evaluation.
